# Predictors of Visceral Infectious Aneurysms in Patients with Infective Endocarditis and Systemic Embolization

**DOI:** 10.3390/jcdd12020057

**Published:** 2025-02-04

**Authors:** Monique Boukobza, Emila Ilic-Habensus, Xavier Duval, Jean-Pierre Laissy

**Affiliations:** 1 Department of Radiology, Bichat-Claude Bernard Hospital, Assistance Publique-Hôpitaux de Paris, 75018 Paris, France; laissyjp.aphp@gmail.com; 2 Clinical Investigation Center, Hopital Bichat-Claude-Bernard, Assistance Publique-Hôpitaux de Paris, 75018 Paris, France; emila.ilic-habensus@aphp.fr (E.I.-H.); xavier.duval@aphp.fr (X.D.); 3 INSERM Clinical Investigation Center 007, 75015 Paris, France; 4 INSERM U738, Paris University, 75016 Paris, France; 5 INSERM U1148, Paris University, 75018 Paris, France

**Keywords:** infective endocarditis, visceral embolic events, visceral infectious aneurysms, spleen infarct, kidney infarct

## Abstract

Background: To study whether infective endocarditis patients (IE-patients) with visceral embolic events (VEEs) at admission are at greater risk of developing visceral infectious aneurysms (VIAs) in left-sided infective endocarditis (LSIE) patients. Methods: We compared the data of prospectively collected 474 consecutive LSIE-patients (2005–2020) with and without VIAs. A whole-body-CTA was part of the initial work-up for all patients. Results: A total of 24 patients (5.1%) with VIA were included, of whom 19 (79.2%) had at least one VEE, compared to a proportion of 34% (*p* < 0.001) in IE-patients without VIAs. Both groups also differed in terms of vegetation size (>15 mm: 48% vs. 18%, *p* < 0.001), microorganisms, *Streptococcus* spp. (68.5% vs. 42%, *p* = 0.003), rare microorganisms (36% vs. 8.3%, *p* < 0.001) and concomitant extra-visceral infectious aneurysms (42% vs. 12.8%, *p* < 0.001). Cardiac surgery was performed in 21 patients (87.5%) and in-hospital mortality occurred in 2 (8%). Conclusions: This study shows a different profile of VIA–LSIE patients compared to LSIE-patients without. *Streptococcus* species were the most frequent causal agents. Our study indicates that the presence of VEEs in LSIE-patients could suggest an increased risk of VIA. This study also shows the need for further abdominal-CTA in all cases of left sided IE to detect asymptomatic visceral aneurysms.

## 1. Introduction

Despite improvements in the diagnosis and treatment of infective endocarditis (IE), embolic events (EEs) may occur, because they are intrinsic to the pathophysiology of IE. They are still a relatively common complication (13–49%), especially in patients with left-sided infective endocarditis (LSIE) [1,2]. Most of these EEs, for the most part in the brain, are produced in the first 2 to 4 weeks, and decrease after successful anti-biotherapy [1].

Recent studies reported that vegetation size (>10 mm), more than vegetation mobility, the nature of the affected valve, i.e., anterior leaflet of the mitral valve [3,4,5], and the causative agent (*Staphylococcus aureus*, *Candida*, and *Haemophilus*, *Aggregatibacter*, *Cardiobacterium*, *Eikenella*, *Kingella* (HACEK) microorganisms) predispose the occurrence of emboli [6,7,8]. In the abdomen, the most frequent LSIE–EEs are in the spleen and in the kidneys, more rarely the liver, producing an infarction that sometimes can evolve into an abscess formation [9].

However, despite the relative frequency of EEs seen on abdominal imaging in LSIE, the association of these visceral EEs (VEEs) and visceral infectious aneurysms (VIAs) has not been analysed [10,11]. We observed a higher prevalence of VEEs in IE patients with VIAs. Thus, the primary endpoint of this study was to compare the incidence of VEEs associated with VIAs in LSIE patients to those without, in order to guide the clinical decision-making process. The secondary objectives were to describe the factors associated with VIAs in LSIE-patients, the clinical implications and the optimal management strategies, including body-CT-angiography (body-CTA).

## 2. Methods

### 2.1. Study Design

The data, prospectively collected, of 474 consecutive patients with definite IE (2005–2020)—identified based on the European Society of Cardiology (ESC) 2023 criteria [12]—were retrospectively reviewed. All patients underwent plain, then dual phase contrast-enhanced (arterial and portal venous phases) CTA acquisitions.

Data for LSIE-patients were compared between those with VIAs and those without.

Patient enrolment included: 1—Definite LSIE (ESC, 2023) [12]; 2—Radiological characteristics consistent with visceral infarct on initial CTA, performed at the acute phase (1–3 days, mean: 2 days); 3—VIA diagnosed on digital subtraction angiography (DSA); 4—age > 18 years. Patients with incomplete data and inconclusive imaging were excluded.

### 2.2. Data Collection

Demographic data, comorbidities, predisposing conditions for IE, background, (cardiac lesions, vegetation size and mobility), bacteriological and radiological features, IE-treatment and IE-complications (embolic events, infections, extra-visceral infectious aneurysms) and in-hospital mortality of IE-patients were analysed via review of the electronic medical records. All imaging examinations were assessed by 2 experienced reviewers in vascular imaging (JPL; MB). Cardiac imaging included TTE and TEE in all patients.

### 2.3. Definitions

The length used for valve vegetations is the maximum value of measurements in different planes. Vegetations are considered mobile, whether this mobility is low, moderate or severe.

Emboli were defined as septic splenic, renal, hepatic or lung emboli, infectious aneurysm, cerebral ischemia or bleeding, cerebral abscess and peripheral major vascular emboli. Visceral infarcts, i.e., infarcts of the spleen, the kidneys and the liver, are defined as a triangular or trapezoidal area of varying size, wedge-shaped, and of low density on CT-scan, that do not enhance after contrast. Infarcts within the spleen and the kidneys have a predominantly peripheral location.

A VEE and a VIA were considered as symptomatic when patients presented with symptoms related to these entities.

Cardiac surgery was defined as emergent (≤24 h), urgent (>1 <8 days), early (8–21 days) and late (>22 days).

Rare agents were defined as representing less than 1% in the overall IE cohort studied. 

### 2.4. Ethics

The study was conducted in accordance with the Declaration of Helsinki. This retrospective study was approved by the institutional review board with waiver of informed consent.

### 2.5. Statistical Analysis

All statistical analyses were performed using SPSS version 25.0 (SPSS Inc., Chicago, IL, USA). For all continuous variables, data were reviewed for normality and. if normally distributed, the independent t test was used. If variables were ordinal or not normally distributed, the nonparametric Mann–Whitney U test was used. Chi-squared or Fisher’s exact tests were used for categorical variables.

Post hoc analyses were performed after excluding those with a preexisting clinical diagnosis of endocarditis, previous ischemic events, or known IA at study entry. This was to establish whether any findings in the cohort as a whole were driven by patients with these diagnoses, which are associated with IA. Statistical analyses were performed using Graphpad Prism (Version 9).

The association between abdominal IAs and other IE characteristics and complications including patient status and comorbidities was analyzed using binary logistic regression, adjusting for sex and age. Univariate and multivariate logistic regression analyses were used to evaluate the correlation between these two groups of patients. The subsequent multivariable linear regression analyses were performed by simultaneously entering the explanatory variables with *p*-values < 0.10 in the bivariate analyses.

Results were expressed as odds ratios (ORs), with 95% CIs for presence of abdominal IAs compared to IA-free IE patients. Values of *p* < 0.05 were considered statistically significant, and both ORs and 95% CIs were also reported.

## 3. Results

**Figure 1:**
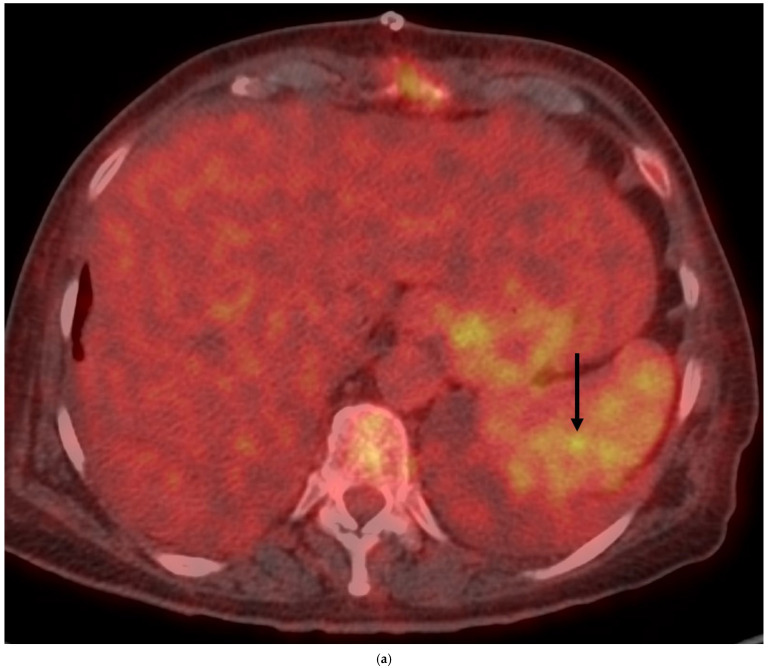

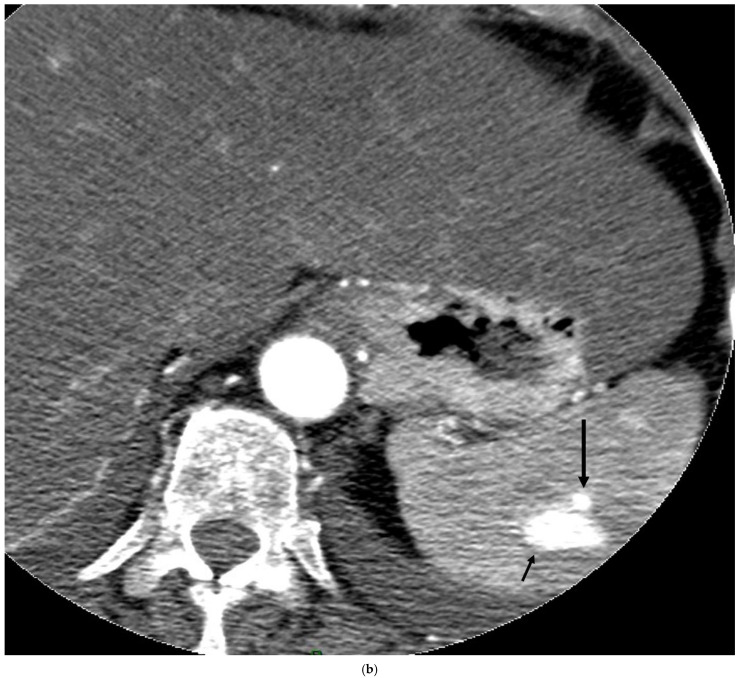

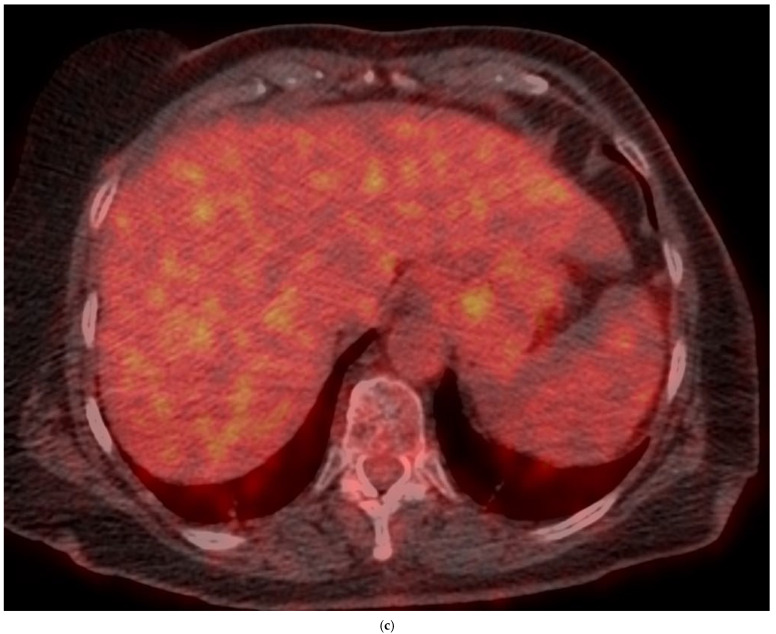
Splenic infectious aneurysm in a *Streptococcus gallolyticus* infectious endocarditis. A 66-year-old woman was admitted for *Streptococcus gallolyticus* native aortic valve infective endocarditis (IE) with vegetations, valve perforation, severe aortic regurgitation, and annular abscess, which required emergent valve replacement and annular reconstruction. Work-up at admission showed multiple splenic infarcts on abdominal CT-angiography (CTA). Whole-body ^18^F-FDG-PET/CT showed intense uptake of an intrasplenic 6 mm-rounded area ((**a**), short arrow). Abdominal CT-angiography (CTA), 4 days later, showed an enhancing lesion consistent with a splenic aneurysm and a contiguous small hematoma ((**b**), long arrow). CTA and PET (**c**) follow-up showed the disappearance of both aneurysm and hematoma. Nine years later she is doing well.

**Figure 2:**
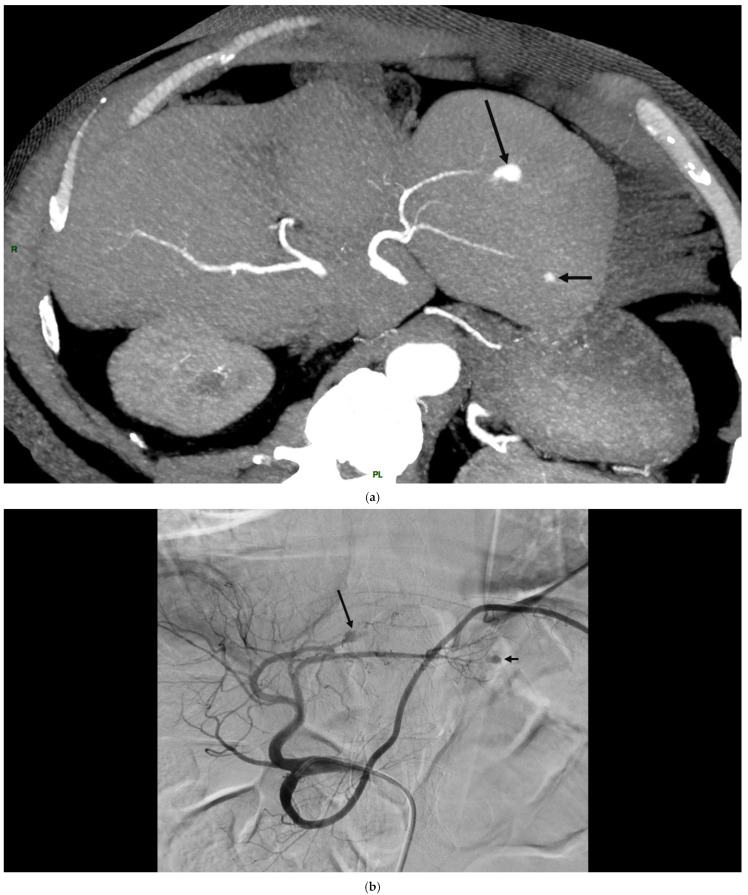

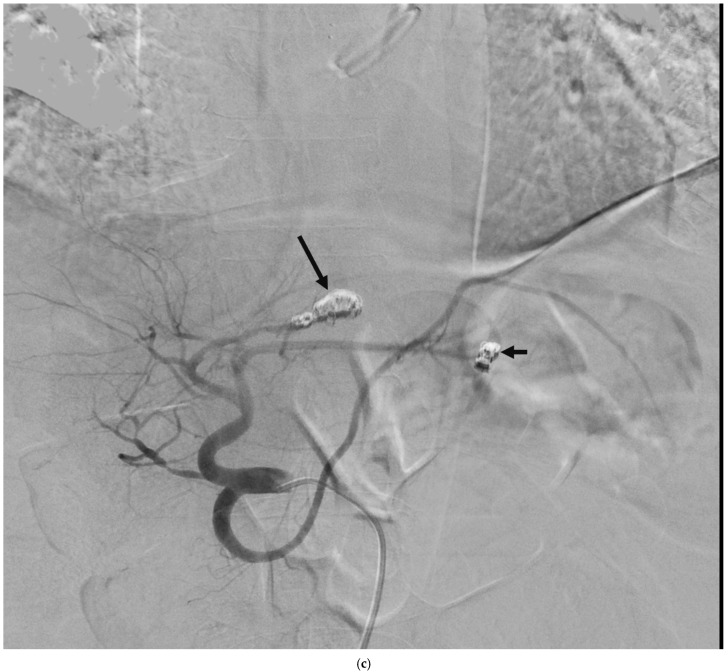
Two hepatic artery infectious aneurysms in a *Streptococcus sanguinis* infectious endocarditis. A 53 year-old man, presented a *Streptococcus sanguinis* infectious endocarditis on Bentall tube, and concomitant splenic infarct and ruptured intracranial aneurysm. Abdominal CT-angiography (CTA) showed two distal and fusiform infectious aneurysms of the left hepatic artery (left liver, segment II) of respectively 12 ((**a**), long arrow) and 4 mm ((**a**), short arrow) in diameter. These aneurysms were evidenced on selective angiography (respectively, (**b**), long arrow and short arrow). Both were excluded by coil embolization (respectively, (**c**), long arrow and short arrow).

**Figure 3:**
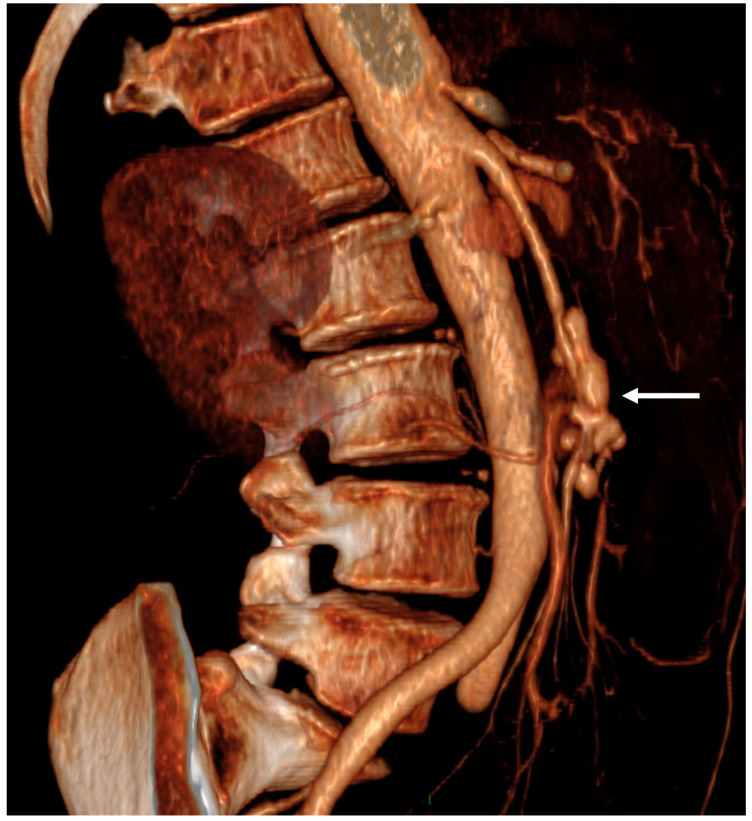
Superior mesenteric infectious aneurysm in a *Streptococcus gordonii* infectious endocarditis. A 55-year-old woman was admitted for a two-month history of fever, fatigue, arthralgia and worsening abdominal pain for one week. Her medical history included hypertension. Abdominal examination at admission showed non-tender abdomen. Transthoracic and transesophageal echocardiography (TTE/TEE) revealed 2 mitral valve vegetations (14 and 25 mm) and severe regurgitation. Blood cultures grew *Streptococcus gordonii*. Antimicrobial therapy with amoxicillin and levofloxacin was initiated. Abdominal CT-angiography (CTA) showed a great lobulated aneurysm (55 mm in maximum diameter) of the superior mesenteric artery (volume rendering, VR, arrow).

From a total of 474 IE patients (main cohort, 2005–2020), we identified 24 (5.1%) patients presenting with a VIA: splenic (n = 6), hepatic (n = 8), superior mesenteric (n = 9) and renal (n = 1) arteries. All the VIAs were diagnosed by CTA and confirmed in all cases by DSA (Figure 1, Figure 2 and Figure 3). VIAs involved one single organ in each patient.

Table 1 shows the baseline characteristics of patients with VIA and those without.

## 4. Clinical Characteristics of VIAs-Patients

VIAs were silent upon arrival in 15/24 (62.5%) and another became symptomatic at day 12 of admission.

Among VIA–IE patients, 19/24 (79.2%) had at least 1 VEE (*p*: 0.0001). Most commonly seen were splenic infarctions (n = 18, 75%), followed by kidney infarction (12, 50%). Both spleen and kidney infarct were observed in half of patients. Liver infarction (n = 2, 8.3%) was associated with a splenic infarct in one patient and with both splenic and kidney infarcts in another one. Six (25%) patients had only a splenic infarct location.

### 4.1. Cardiac Lesions

Among patients with VIAs, IE involved the aortic valve in 8 (33.3%) and mitral-aortic in 3. A native valve infection was detected in 15 (62.5%: mitral 10, aortic 3; mitral-aortic 2), and 9 (37.5%) had prosthetic valve infection (mitral 3, aortic, 5; mitral-aortic 1). A device lead infection was observed in 1 patient. Abscesses were present in 2 patients (8.3%).

### 4.2. Associated Lesions (Table 1)

Seventeen (70.8%) patients had additional EE. Patients with VIAs had significantly more associated extra-visceral IAs than non-VIAs IE patients. These concerned mostly cerebral arteries distally (n = 8, 33%) and limb arteries (n = 3). One patient had two extra-visceral IAs.

### 4.3. Comparison of Left-Sided Infective Endocarditis with and Without Visceral Infectious Aneurysms

Both groups were statistically indifferent in terms of sex, age, cardiac abscess, valve perforation extra-visceral emboli, pre-existing conditions for IE, i.e., previous IE, and valvular predisposition (Table 1).

The various features of IE patients are presented in Table 2.

**Figure 4:**
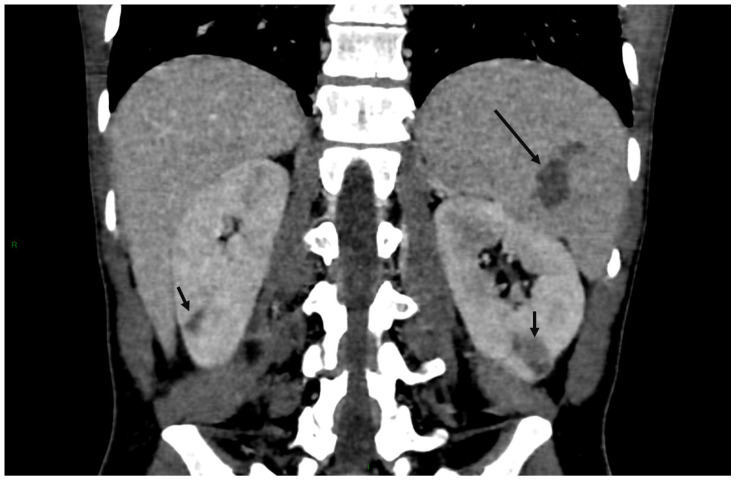
Infarcts of the spleen and both kidneys in a *Streptococcus oralis* infectious endocarditis complicated by a hepatic infectious aneurysm. Abdominal CT scan without contrast shows both splenic infarction (long arrow) and infarcts of right and left kidneys (short arrows).

Those with abdominal IAs were more likely to have large valve vegetations. They had more visceral infarctions (Figure 4), intracranial or extracranial infectious aneurysms, mitral valve location, prosthetic valves, and comorbidities (IVDU, alcohol consumption). Diabetes did not predict abdominal IA. In multivariable logistic regression analysis (Table 2), in which each predictor was considered separately, the presence of hepatic infarction (OR, 13.64, 95% CI, 1.19–156.74), other infarct locations excluding the spleen and the kidneys (OR, 12.34; 95% CI, 4.02–37.9), kidney infarction (OR, 7.58; 95% CI, 2.81–20.42), spleen infarction (OR, 6.88; 95% CI, 2.43–19.49), intracranial IAs (OR, 5.24; 95% CI, 1.79–15.33) and large vegetations (OR, 2.7; 95% CI, 1.13–6.47) were independently associated with abdominal IA.

In the multiple linear regression analysis, the vegetation size and type of valve involved with *p*-values < 0.10 in the bivariate analyses were used as explanatory variables and the IA was used as the outcome variable. As a result, vegetation size (*p* < 0.001) and aortic valve involvement (*p* = 0.018) were suggested to be the significant characteristics in relation to the presence of visceral IA.

The other factors, such as multiple or prosthetic valve involvement, previous IE, diabetes, spondylodiscitis and streptococcus, were not predictors for IA.

### 4.4. Treatment and Outcome

In all patients but three (n = 21, 87.5%), cardiac surgery was indicated and was emergent (n = 3) urgent (n = 8), early (n = 7) and late (n = 3). It consisted in biological valve replacement (VR) in 19 (79.2%) and in mechanical VR in 3 (12.5%). Despite non-significance, the rate of heart surgery was higher than in non-VIAs patients (87.5% vs. 75%).

In-hospital mortality was similar in the two groups of patients. It occurred in two patients (8%) in the VIAs group: one patient died after emergent cardiac surgery for congestive heart failure, within 24 h, from multiorgan failure; the other died from cerebral hemorrhage which occurred after vascular surgery of ruptured tibio-fibular IA in a Marfan disease’ patient. In-hospital mortality occurred in 30/450 (6.7%) in the non-VIA–IE patients.

In total, coil embolization of VIAs was prescribed in 12 patients (50%), whatever the location of the VIA, either before (6/12) or after cardiac surgery (6/12). Among patients who underwent coil embolization after surgery, in one case the VIA was delayed. VIAs regressed under anti-biotherapy alone in 9/24 (37.5%).

Surgical treatment (ligation-graft or resection-graft) was carried out post-cardiac surgery in three patients (1.25%) and concerned superior mesenteric artery-IAs in all these cases.

## 5. Discussion

To our knowledge, this is the first cohort describing factors associated with VIAs in patients with LSIE. VIAs are a rare complication of IE and only a few series have been reported [10,11], thus their profile has not been clearly described until now. Our main finding is that the most predictive factors of abdominal IA were spleen, kidney and liver infarcts, intracranial aneurysms and valvular vegetation size (>15 mm predominantly on the mitral valve, in IVDU patients. On the contrary, there was no significant relationship between abdominal IA and multiple or prosthetic valve involvement, previous IE, diabetes and streptococcus.

Conversely, as for the proportion of LSIE-patients with VEEs and without VIAs (34%), their distribution—mostly located in the spleen and the kidney—was in concordance with published findings of the post-antibiotic era [6,13,14,15,16]. In our case, differences with reported series, when occur, are explained by the lack of routinely performed CTA to screen intra-abdominal embolisms, or because studies consider only symptomatic patients.

Secondly, LSIE patients with VIAs display different characteristics compared to LSIE patients without, one being related to *Streptococcus* spp. predominance.

It is well established that a vegetation size greater than 10 mm is associated with all EEs and with new EEs [15,16,17] and that the risk is even higher for very large vegetations (≥15 mm) and mobile vegetations [6,7,8,9,10,11,12,13,14,15,16,17,18].

In 2020, the American College of Cardiology/American Heart Association (ACC/AHA) considered surgery for patients with left-sided IE who present mobile vegetations >10 mm, with or without associated EEs, but with a low strength of recommendation (class IIb) [19].

We observed that vegetation size >15 mm seems to be strongly associated with VIAs, in keeping with all studies, especially recent ones [2,16,17,18,19,20]. These studies demonstrated that the occurrence of EEs was related to the vegetation size in patients with aortic valve or mitral valve IE but not with their number and mobility. Parra et al. [20], based on 147 LSIEs, showed that patients with VEEs had larger vegetations, but also non-abdominal emboli, among other lesions.

Most EEs in IE have been associated with *Staphylococcus aureus*, *Candida* and HACEK microorganisms [3,7]. Recent studies underlined that staphylococcal endocarditis is a risk factor for overall EEs [2,3,4,5,15,16,17]. However we did not observe an association between VIAs and *Staphylococcus aureus* infection. On the contrary, our study showed that *Streptococcus* spp. are most often the causal microorganisms in VIA–IE, as seen in previous studies, which concerned only a few intracranial IA in IE patients [21,22,23], and in more recent and large studies and reviews [24,25,26,27]. Indeed, *Streptococcus* spp. are preponderant causal agents for intracranial IAs in IE, being almost twice as frequent in this context. Our findings are in line with Parra et al. [20], who found in a study based on 147 LSIE patients that, among patients with spleen-kidney-liver EEs (34%), *Streptococcus* species were the most prevalent microorganisms (40%). In Calderon-Parra et al. [28], based on 4548 definite IE patients, 85 presenting an IA, *Staphylococcus aureus* was not associated with intracranial nor extracranial IA.

The problematic of the microorganisms involved in VIA–IE has not yet been addressed previously: our findings suggest that the causative agents frequently associated with IAs in IE, in both intracranial and visceral locations, are the *Streptococcus* spp.

Papadimitriou-Olivgeris et al. [5] recently reported that intracardiac abscesses are risk factor for EEs. In our series of VIAs, we identified intracardiac abscesses in only two (8%) cases. Lecomte et al. [29], in a study based on 522 IE-patients, found that whole-body CT findings affected diagnosis and treatment of IE in a very small proportion of asymptomatic patients and underlined the limitations due to the high risk of acute kidney injury (AKI). However, the percentage of patients with VIAs in our main cohort is much more significant than that found by Lecomte et al.: 24/474 (5.1%) vs. 8/522 (1.5%) [29]. Since Lecomte et al. [29] include both definite IEs and possible IEs in their study, this may explain, at least in part, their conclusions, which differ from ours.

A plausible explanation for the association of VIAs and VEEs could be that the microbial load in emboli that lead to arterial occlusions can potentially, at the same time, injure the arterial wall, which may lead to the formation of an IA.

## 6. Limitations and Strengths

Limitations of this study relate to its retrospective and observational nature as well as moderate sample size and single-center, which might have an influence on some of the results obtained. This study is not exempted from selection bias (tertiary hospital, recruitment) because our series may not represent the situation of all LSIE-patients, where complicated cases of IE might be over-represented and occult ones underrepresented.

Thus, although we demonstrate a strong and novel association between VEEs and VIAs, our research design does not allow us to infer causality. Nonetheless, our findings may provide additional insight into the association between VIAs and VEEs that will require elaboration in future prospective studies.

Besides, logistic regression does not consider relations of adverse events with time. VIAs require some time to develop and may well be more easily detected in cases of more prolonged intervals from the start of the infectious process, with a consequently increased probability of embolic events. Indeed, it is difficult to define time intervals retrospectively because the exact moment when IE starts is impossible to determine.

Strengths of our study relate to inclusion of consecutive patients with available CTA at admission.

## 7. Conclusions

Our study shows that LSIE-patients with VEEs are more likely to have VIAs. Thus, the presence of VEEs in LSIE-patients could suggest an increased risk of VIA. Consequently clinicians should consider CTA in VEE–IE patients to help better target those with potential associated VIA. Furthermore, since these VIAs may be clinically silent, CTA has a key role to play in their diagnosis.

Nonetheless, due to the sample size of this study, our results should be corroborated by larger observational studies. Individualized decision-making among the endocarditis team in reference centres is a key issue in the management of LSIE with VIAs.

## Figures and Tables

**Table 1:** Characteristics of Infective Endocarditis (IE) patients with visceral infectious aneurysm vs. IE-patients without.

	VIAs n = 24 (%)	No VIAs s n = 450 (%)	*p* (Value)
Age (years), median, range	49.5 [19–75]	51 [18–83]	
Male Gender	17 (70.8)	360 (80)	0.3
Cardiac conditions for IE			
Prosthetic valve	8 (33.4)	85 (19)	0.11
Previous IE	3 (12.5)	32 (7)	0.24
Previous cardiopathy	3 (12.5)	99 (23)	0.32
Atrial fibrillation	1 (4.2)	32 (7)	1
Antithrombotic therapy			
Prior anticoagulant therapy	3 (12.5)	67 (15)	1
Antiplatelet drugs	0	33 (7.3)	1
Valve			
Native valve	15 (62.5)	342 (76)	0.15
IE location			
Mitral	13 (54.1)	202 (45)	0.41
Aortic	7 (29.2)	211 (47)	0.5
Both	4 (16.7)	32 (7)	0.1
Prosthetic valve	9 (37.5)	108 (24)	0.15
Valve vegetations	20 (84)	284 (65)	0.08
Vegetations >10 mm	18 (68)	162 (36)	<0.001
Vegetations >15 mm	13 (48)	80 (18)	<0.001
Vegetation mobility (% among Vegetations)	9 (45)	112 (25)	0.23
Abscess	2 (8.3)	54 (12)	0.5
Visceral emboli			
Nb patients having at least 1 VIA	19 (79.2)	147 (34)	<0.001
Spleen	18 (75)	135 (30)	<0.001
Kidney	12 (50)	45 (10)	<0.001
Hepatic	2 (8.3)	10 (2.2)	0.12
Extravisceral emboli			
Brain (CMBs not included)	10 (42)	157 (35)	0.37
Pulmonary	3 (12.5)	54 (12)	0.69
Limbs	2 (8,3)	14 (3.2)	0.2
Coronary	2 (8.3)	8 (1.8)	0.09
Associated Infectious Aneurysms (by patients)	10 (42)	57 (12.8)	<0.001
Abscesses total	6 (25)	62 (13.7)	0.13
Brain	2 (8.3)	25 (5.6)	0.64
Splenic	4 (16.7)	34 (7.3)	0.11
Spondylodiscitis	4 (16.7)	32 (7)	0.1
Microorganisms *			
*Streptococcus* spp.	15 (68.5)	189 (42)	0.003
*Staphylocccus* spp.	5 (20.8)	153 (34)	0.27
*Enterococcus faecalis*	2 (8.3)	32 (7)	0.68
Rare microorganisms	9 (36)	37 (8.3)	<0.001
Preexisting conditions			
Comorbidities			
Diabetes melitensis	1 (4.2)	63 (14)	0.35
Hemodialysis/end-renal failure	0	14 (3.2)	1
Malignancy	1 (4.2)	32 (7)	1
IVDU	2 (8.3)	22 (5)	0.34
HIV	0	14 (3.2)	1

VIAs: with visceral infectious aneurysms; IE: Infective Endocarditis; CMBs: Cerebral microbleeds; IVDU: Intravenous drug use. * One patient had 2 microorganisms, and 1 had 5 microorganisms: 29 microorganisms in total.

**Table 2:** Multivariable linear regression for clinical, microbial and imaging predictors of infectious aneurysms.

	OR	95 CI	*p* Value
Spleen infarction	6.88	2.43–19.49	0.023
Kidney infarction	7.58	2.81–20.42	0.002
Liver infarction	13.64	1.19–156.74	0.004
Other infarctions	12.34	4.02–37.9	<0.001
Intracranial IAs	5.24	1.79–15.33	0.024
Extracranial IAs	1.43	0.29–7.08	0.517
Prosthetic Valve	2.3	0.89–5.91	0.066
Multiple Valves	0.49	0.14–1.75	0.148
Vegetation > 15 mm	2.7	1.13–6.47	0.045
Mitral valve	1.12	0.47–2.66	0.042
Aortic valve	0.31	0.13–0.77	0.106
Previous IE	1.3	0.35–4.86	0.071
Streptococcus	1.9	0.79–4.56	0.197
Diabetes	0.34	0.04–2.7	0.19
IVDU	1.52	0.4–5.77	0.004
Alcohol	0.91	0.31–2.6	0.016

OR, odds ratio, CI, confidence interval, IA infectious aneurysm, IE infectious endocarditis, IVDU intravenous drug user.

## Data Availability

Data are available from the corresponding author upon reasonable request.
